# Promotion of Tumor Invasion by Tumor-Associated Macrophages: The Role of CSF-1-Activated Phosphatidylinositol 3 Kinase and Src Family Kinase Motility Signaling

**DOI:** 10.3390/cancers9060068

**Published:** 2017-06-18

**Authors:** Amy R. Dwyer, Eloise L. Greenland, Fiona J. Pixley

**Affiliations:** School of Biomedical Sciences, The University of Western Australia, 35 Stirling Highway, Crawley, WA 6009, Australia; ardwyer@umn.edu (A.R.D.); 20369309@student.uwa.edu.au (E.L.G.)

**Keywords:** macrophage motility, colony-stimulating factor-1, phosphatidyl-inositol-3-kinase, Src family kinases, paracrine interaction.

## Abstract

Macrophages interact with cells in every organ to facilitate tissue development, function and repair. However, the close interaction between macrophages and parenchymal cells can be subverted in disease, particularly cancer. Motility is an essential capacity for macrophages to be able to carry out their various roles. In cancers, the macrophage’s interstitial migratory ability is frequently co-opted by tumor cells to enable escape from the primary tumor and metastatic spread. Macrophage accumulation within and movement through a tumor is often stimulated by tumor cell production of the mononuclear phagocytic growth factor, colony-stimulating factor-1 (CSF-1). CSF-1 also regulates macrophage survival, proliferation and differentiation, and its many effects are transduced by its receptor, the CSF-1R, via phosphotyrosine motif-activated signals. Mutational analysis of CSF-1R signaling indicates that the major mediators of CSF-1-induced motility are phosphatidyl-inositol-3 kinase (PI3K) and one or more Src family kinase (SFK), which activate signals to adhesion, actin polymerization, polarization and, ultimately, migration and invasion in macrophages. The macrophage transcriptome, including that of the motility machinery, is very complex and highly responsive to the environment, with selective expression of proteins and splice variants rarely found in other cell types. Thus, their unique motility machinery can be specifically targeted to block macrophage migration, and thereby, inhibit tumor invasion and metastasis.

## 1. Introduction

Macrophages are found throughout the body where they contribute to tissue development, remodeling and homeostasis [1]. These multifunctional cells can be considered accessory or housekeeping cells, as they support the diverse needs of parenchymal cells in different tissues [2]. Hence, resident macrophages survey the local environment, secrete trophic factors, phagocytose cells and debri, and carry out repairs to maintain homeostasis. Their distribution within each tissue is also highly structured to enable functional coordination within the neighborhood and their trophic and repair capacities are tightly regulated to ensure activation occurs only when appropriate [2]. To integrate seamlessly into different tissue environments and carry out tissue-specific functions, as well as general housekeeping functions, macrophages undergo a remarkable degree of transcriptional reprogramming in response to local cues [3]. This plasticity can be problematic when regulatory mechanisms fail in different disease states, as their developmental functions can be reactivated and their housekeeping functions subverted, to promote disease progression in conditions ranging from atherosclerosis and arthritis, to cancer [4,5].

**Figure 1:**
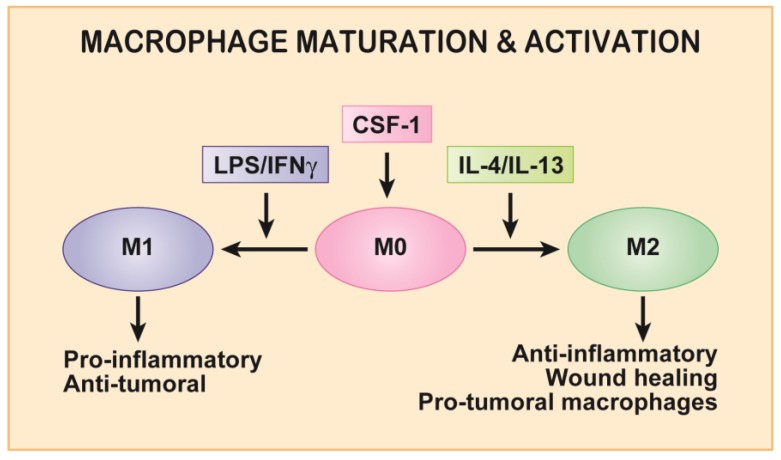
Colony-stimulating factor-1 (CSF-1) induces macrophage precursors to mature into adherent, motile baseline macrophages (M0). M0 macrophages are then activated into pro-inflammatory macrophages by IFNγ and LPS or anti-inflammatory, pro-tumoral macrophages by IL-4 or IL-13. Definitions: LPS, lipopolysaccharide; IFNγ, interferon-γ; IL, interleukin.

Despite their functional complexity, macrophages are frequently categorized in a binary manner as either classically activated, pro-inflammatory (M1) or alternatively activated, anti-inflammatory (M2) macrophages, following exposure to interferon-γ (IFN-γ) and bacterial infections or interleukin (IL)-4 and IL-13, and parasitic infections, respectively (Figure 1) [6].

As M2 activation can also be caused by other stimuli, M2 macrophages have been further stratified in terms of homeostasis, host defense, wound repair and immune regulation [7], although recent results indicate that activation phenotypes are far more complex than that which can be accommodated by a linear continuum [3,8]. Moreover, different macrophage populations co-exist within a single tissue and, particularly in pathological conditions where the microenvironment can be highly variable such as in tumors, macrophages may develop mixed phenotypes [5]. Solid tumors frequently contain large numbers of tumor-associated macrophages (TAMs), which contribute to their development and progression [5,9]. Indeed, a tumor can be thought of as an organ with its own integral resident macrophages, which have been groomed by the tumor cells to provide them with trophic factors for growth and vascular supply, as well as aiding them to resist immune attack and cancer therapies [5,9].

Interstitial migration is central to the macrophage function as they patrol tissues to carry out their many developmental and housekeeping duties [10]. Live imaging of mouse mammary tissue showed macrophages crawling along and between collagen fibers at rates of approximately 5 µm/min [11]. Colony-stimulating factor-1 (CSF-1), the macrophage growth factor, was originally demonstrated to signal to survival, proliferation and differentiation of cells of the mononuclear phagocytic (MNP) lineage, including monocytes and macrophages [12]. It is found at biologically active concentrations throughout the body and, importantly, is considered the primary cytokine in macrophage development [12]. Consequently, an international collective of macrophage biologists determined that macrophages grown in CSF-1 should be labeled baseline (M0) macrophages that subsequently may be activated by other factors in the microenvironment, such as IFN-γ or IL-4 (Figure 1) [13]. Hence, CSF-1 is considered a maturation rather than an activation factor. Consistent with this, CSF-1 stimulates differentiation of mouse and human monocytes into mature, adherent macrophages after which it remains an important survival factor [10,13,14]. CSF-1 is also a critical regulator of macrophage motility, i.e., it is a macrophage chemokine [15,16,17,18]. Considering the importance of interstitial migration for macrophages in both health and disease, regulation of this process by CSF-1 deserves more attention. This review focuses on motility signals transduced by the CSF-1R upon activation by CSF-1, and how these signals might be targeted to treat disease, particularly cancer.

## 2. Macrophage Motility and CSF-1/CSF-1R Axis in Development and Homeostasis

Studies of organisms deficient in CSF-1 or its receptor have revealed the importance of macrophages in matrix remodeling, branching morphogenesis and phagocytosis of dying cells during organogenesis [11,19,20,21,22,23,24]. Consistent with the central role of CSF-1 in the regulation of motility, a loss of function in CSF-1R mutation in zebrafish specifically stops primitive yolk sac-derived macrophages from migrating into embryonic tissues [25]. The microglial population is particularly affected by this migratory defect. Similarly, inactivation of *Csf1* in the *osteopetrotic* mouse (*Csf1^op/op^*) produces multiple congenital abnormalities, many of which may result from failure of primitive macrophages to migrate into and populate developing tissues [23,26,27]. Notably, CSF-1 is secreted by mammary epithelial cells to recruit macrophages into the developing mouse mammary gland, where they migrate along and remodel collagen fibrils to enable terminal end bud lengthening [11]. Motility may also be required for their role in branching morphogenesis and vascular anastomosis [15]. Recently, mutations in the human CSF-1R have been shown to underpin an autosomal dominant form of early onset dementia, hereditary diffuse leukoencephalopathy with spheroids (HDLS), which predominantly affects frontal lobe white matter [28,29]. Interestingly, the CSF-1R mutations all occur in the kinase domain and lead to reduced expression and phosphorylation of the receptor, indicating that CSF-1R haploinsufficiency is sufficient to cause HDLS, which is also known as adult-onset leukodystrophy, with axonal spheroids and pigmented glia (ALSP) [30,31]. Importantly, primitive macrophage-derived microglia are CSF-1 dependent, and both their morphology and distribution in the frontal lobe white matter are aberrant in patients with HDLS, suggesting microglial motility may play a role in the pathogenesis of this condition [30,32]. The neurologically restricted phenotype of HDLS, which is consistent with neuro-developmental findings in a Csf1r^+/−^ mouse model, suggests that microglial CSF-1R signaling is particularly critical for normal cortical white matter development and maintenance [31]. Finally, the central role of migratory macrophages in the patterning of adult zebrafish stripes has recently been demonstrated [33,34], which leads to the aberrant pigmentation of *Csf1r* mutant zebrafish [25]. 

In the adult organism, resident macrophages contribute to the homeostasis of their tissues of residence through immune surveillance and wound healing as well as through trophic and phagocytic activity. Macrophages may form up to 20% of tissue mass in organs such as the liver [20,35] and, as mentioned earlier, they form complex networks within the tissues to efficiently patrol and clean the neighborhood. Macrophages play a pivotal role in metabolic homeostasis and energy regulation through their integration in the liver, pancreatic islets and adipose tissue, while in the spleen, they phagocytose senescent erythrocytes and recycle essential components such as iron, and microglia are important for learning and memory [2,36,37,38]. Hence, different populations of macrophages have essential developmental and homeostatic functions in all tissues, helping parenchymal cells to function normally and respond appropriately to variable demands. CSF-1 is important for the maintenance of most, if not all, of these populations, although additional cytokines also regulate macrophage development and behavior. The most notable of these is IL-34, which was discovered following the demonstration that the Csf1r^−/−^ mouse was more severely affected than the Csf1^−/−^ mouse [39,40]. CSF-1 is the dominant cytokine of the two as the main effects of IL-34 are in the development of Langerhans cells in the skin and some microglial populations and the IL-34^−/−^ mouse is essentially normal [41,42,43]. Macrophage proliferation can also be stimulated by granulocyte macrophage (GM)-CSF or IL-4 [44,45] but macrophages grown in GM-CSF are less adherent and elongated while IL-4-activated macrophages elongate and move faster than those grown in CSF-1 alone [44,46,47,48]. Nevertheless, CSF-1 is considered to be the fundamental growth factor regulating production and differentiation of mature macrophages with an adherent and motile M0 phenotype [13].

## 3. Macrophage Motility and CSF-1 in Cancer

**Figure 2:**
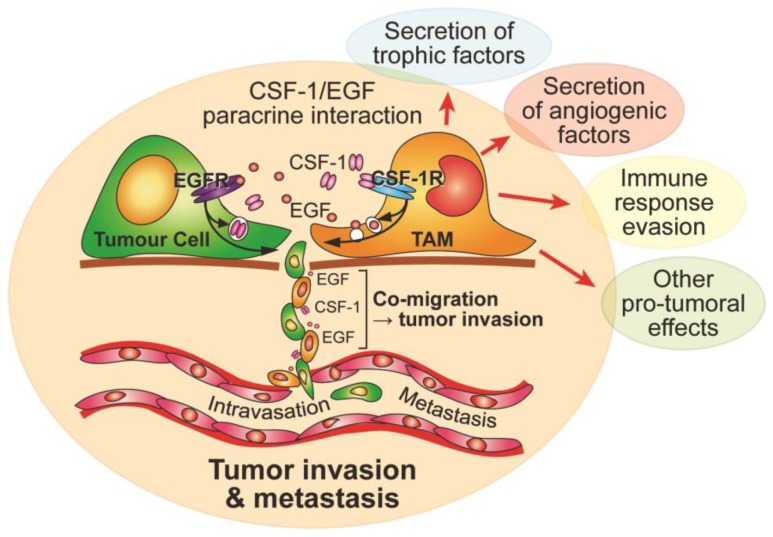
Contribution by tumor-associated macrophages (TAM) to tumor progression with an emphasis on tumor invasion and metastasis. TAMs secrete epidermal growth factor (EGF) and tumour cells secrete CSF-1 to induce a paracrine loop-driven co-migration and invasion of both cell types towards blood vessels.

Due to their functional integration in tissues and organ systems, macrophages are readily subverted by disease mechanisms in those same tissues [49]. They contribute to the development and progression of atherosclerotic plaques [50], obesity and metabolic disease [38], rheumatoid arthritis [51], and neurological disorders such as multiple sclerosis [52]. The most striking pathological involvement of macrophages is their largely deleterious role in the progression of cancer to invasion and metastasis [5,9,12,53]. TAMs are principally derived from circulating monocytes rather than from proliferation of local tissue-resident macrophages [54], and they contribute to a number of cancer hallmarks, including stimulation of tumor cell proliferation and angiogenesis through production of growth factors (Figure 2) [9,12]. However, perhaps the most lethal aspect of their role in cancer progression is their promotion of tumor invasion and metastasis [55]. Macrophage-deficient female CSF-1-deficient mice, when crossed with the mammary tumor-prone Polyoma middle T antigen (PyMT) mouse, rarely develop pulmonary metastases, despite the formation of large numbers of primary mammary epithelial tumors [56]. As mentioned above, CSF-1 is secreted by ductal epithelial cells to recruit macrophages, which are required for normal mammary gland development and homeostasis [7], but it is also secreted by breast cancer cells to recruit TAMs, which promote progression of the cancer to invasion and metastasis [57]. A paracrine interaction between CSF-1-secreting tumor cells and epidermal growth factor (EGF)-secreting TAMs stimulates relay chemotaxis through direct cell-cell contact, leading to invasion of both cell types together towards blood vessels (Figure 2) [5,57,58,59]. Indeed, streams of TAMs and tumor cells have been seen migrating together along collagen fibers within tumors [60]. Accordingly, high TAM densities correlate strongly with metastatic breast cancer, and high CSF-1 levels coincide with dense TAM foci at invading fronts of breast cancers [55,61,62,63]. High TAM counts are associated with a poor prognosis in a number of cancers, including cervical cancer, endometrial cancer, prostate cancer, lung cancer, pancreatic cancer and glioblastoma [64,65,66,67,68,69].

Consistent with the role of CSF-1-dependent, TAM-activated tumor invasion, high CSF-1 levels are also associated with poor prognoses in many cancers, leading to the testing of a variety of anti-CSF-1/CSF-1R therapies to either reduce or reprogram TAMs to treat cancer [70]. Indeed, patients with a rare synovial cell-derived diffuse-type giant cell tumor, which results from a translocation event that causes uncontrolled CSF-1 production by synovial cells with consequent recruitment of huge numbers of macrophages, responded dramatically to administration of an antibody blocking the CSF-1R (emactuzumab) [71]. Furthermore, anti-CSF-1/CSF-1R therapies have produced encouraging therapeutic responses in pre-clinical tumour models, particularly in combination with chemotherapy or radiotherapy [72,73,74], or when used to reprogram tumor-promoting TAMs into anti-tumoral TAMs [69,75]. Hence, a number of clinical trials with small molecule inhibitors or antibodies targeting the CSF-1R are currently underway [9]. However, there is some evidence that CSF-1R blockade may encourage accumulation of deleterious tumor-associated neutrophils [76]. This may be due to the effect of CSF-1R inhibition on anti-tumoral macrophage populations as functionally distinct TAM populations are found within tumours [77]. Therefore, since the metastatic spread of tumors accounts for the vast majority of cancer deaths and TAMs activate tumor invasion and metastasis through paracrine CSF-1/EGF signalling (Figure 2), inhibition of specific CSF-1R signals supporting pro-tumoral TAM behaviour may leave anti-tumoral TAM populations relatively unaffected. Hence, development of therapies targeting TAM motility may be an effective approach to prevent the co-migration and invasion of tumor cells with TAMs.

## 4. Regulation of Macrophage Motility by CSF-1R Signaling

CSF-1 supports survival, proliferation, differentiation and migration in macrophages and the mononuclear phagocytic progenitor cells from which they are derived. Reflecting the chameleon-like ability of differentiating macrophages to adapt to their different tissues of residence, their transcriptome is both highly complex and extremely plastic [3]. Nevertheless, whatever their final tissue of residence, CSF-1 induces their maturation from circulating monocytes into adherent and motile tissue-resident macrophages [10]. Consistent with this notion, CSF-1 induces the expression of adhesion and motility molecules [44,78,79], and stimulates tyrosine phosphorylation of significantly more cellular proteins than does GM-CSF [80].

**Figure 3:**
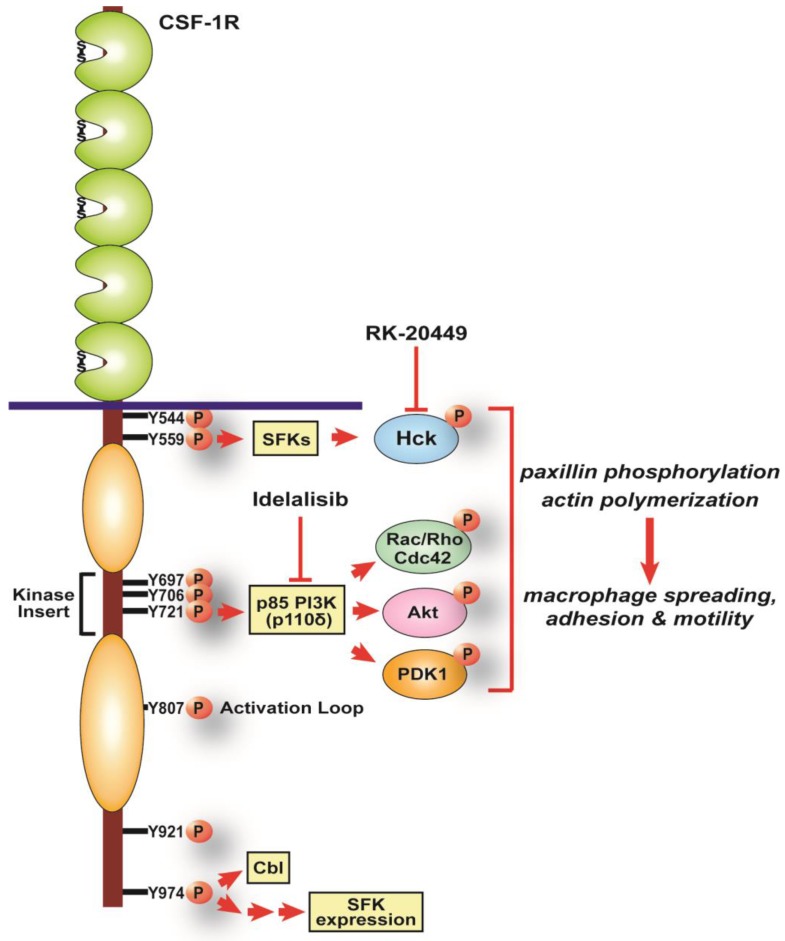
Schematic of the colony-stimulating factor-1 receptor (CSF-1R) and downstream motility signaling. Upon binding of CSF-1, the CSF-1R homo-dimerizes and autophosphorylates up to eight tyrosine residues. Both the pY721 and pY974 motifs have been shown to be important for motility signaling. pY721 directly stimulates macrophage motility through activation of PI3K p110δ, leading to activation of downstream signalling involving the Rho GTPases (Rac, Rho and Cdc42), Akt and PDK1. pY974, which has been demonstrated to bind the E3 ubiquitin ligase, c-Cbl, also indirectly affects motility signalling through regulation of expression of Src family kinases (SFK). Hck, and possibly other SFKs, directly regulate CSF-1-induced motility, likely through integrin-mediated activation of adhesion and actin polymerization.

Like other receptor tyrosine kinases (RTKs), the CSF-1R transduces CSF-1’s effects via a series of tyrosine phosphorylation events (Figure 3) [18,46]. Upon CSF-1 binding, the receptor phosphorylates up to 8 intracellular tyrosine residues and the resulting phosphotyrosine motifs create specific docking sites for phosphotyrosine (pTyr) binding domain-containing proteins. Docked molecules trigger multiple downstream signals to produce and maintain macrophage numbers and functional capacities [18,46]. To tease apart individual pathways mediating the pleiotropic effects of CSF-1, we developed a macrophage cell line system with individual tyrosine-to-phenylalanine mutant CSF-1Rs expressed in macrophages derived from the CSF-1R^−/−^ mouse [44]. The system was used to demonstrate that specific CSF-1R pTyr motifs regulate macrophage morphology, while others are critical for macrophage proliferation and differentiation [44,81]. Two pTyr residues in particular were subsequently shown to regulate macrophage motility, pY721 in the kinase insert domain and pY974 at the C-terminus (Figure 3) [14,79].

Interestingly, the pathways activated by the two pTyr motifs are quite different and shed light on the mechanisms by which CSF-1R signaling regulates motility in macrophages acutely through rapid stimulation of cell ruffling, membrane protrusion and adhesion and in the longer term through stimulation of gene transcription (Figure 3) [10].

## 5. CSF-1R pY721, PI3K Signaling and Macrophage Motility

In macrophages deprived of CSF-1, rapid activation of actin polymerization underpins the dorsal ruffling that becomes visible within a minute of CSF-1 addition [79]. This is swiftly followed by cell spreading as lamellipodia extend circumferentially, supported by the formation of countless dot-like adhesions on the ventral surface of the cell [10,82,83]. Maximal spreading occurs within approximately five minutes [84], after which macrophages polarize, elongate and move persistently towards the source of CSF-1 [16,85]. Y721F mutant CSF-1R-expressing macrophages are deficient in all the CSF-1-stimulated steps leading to motility outlined above, namely, ruffling and spreading, adhesion formation and elongation, resulting in a striking reduction in their motility in vitro and in vivo [79]. Critically, there is also a significant reduction in their capacity to enhance tumor cell invasion in vitro and in vivo [79]. The CSF-1R Y721 motif, when phosphorylated, associates with, and activates, phosphatidylinositol 3-kinase (PI3K), which rapidly produces a pulse of PI 3,4,5-trisphosphate (PIP3) at the cell membrane [79,86,87]. Thus, like other RTKs, the CSF-1R activates class IA PI3Ks via direct association of their p85 regulatory subunit with the preferred pYXXM motif in the RTK which, in the case of CSF-1R Y721, is pYVEM in the kinase insert region (Figure 3). p85 PI3K may also associate indirectly with the receptor either through its association with SFKs at CSF-1R pY559 or with c-Cbl at CSF-1R pY974, but this indirect association is at very low levels and occurs significantly later than the pY721-based association [46,79,87,88].

Class 1A PI3Ks consist of three p110 catalytic isoforms constitutively associated with the regulatory p85 subunit. Macrophages express all three of them; the ubiquitously expressed p110α and p110β isoforms, and the hematopoietically expressed p110δ, and they have non-redundant biological roles [84,89,90]. Together, their activation triggers a plethora of signals to growth, survival, proliferation and migration by inducing the translocation of pleckstrin homology (PH) domain-containing molecules such as Akt/PKB [91,92]. Use of selective inhibitors for each of the three class 1A p110 isoforms has clearly shown that p110δ is the specific isoform triggering CSF-1-induced macrophage motility and invasion [84,89]. p110δ-produced PIP3 activates signaling pathways that are yet to be fully elucidated, but which stimulate two waves of actin polymerization within 5 minutes and a slightly later increase in paxillin phosphorylation to underpin the CSF-1-induced ruffling, spreading and adhesion formation [79,84]. Two PI3K-activated pathways known to influence cell motility are PDK1/Akt and Rac signaling (Figure 3). Although CSF-1-induced Akt phosphorylation is reduced and its kinetics slowed in CSF-1R Y721F macrophages, significant Akt phosphorylation still occurs in these cells in the absence of any PIP3 production [79,84]. This partial uncoupling of motility and Akt activation suggests Akt-independent pathways also regulate pY721-stimulated motility. Likely candidates include PDK1, the S/T kinase responsible for activating Akt but which also activates several isoforms of PKC that may control cell motility [93]. Similarly, CSF-1-based regulation of PH domain-containing guanine nucleotide exchange factors (GEFs) and GTPase activating proteins (GAPs) may also play a role through modulation of the activities of their target Rho family GTPases, Rho, Rac and Cdc42 [10,79]. Rho family GTPases are well known regulators of actin polymerization and cell adhesion downstream of RTKs in many different cell types through their interaction with a variety of effector proteins [94]. Selective expression of individual Rho family GTPase members and their GEFs, GAPs and effector proteins results in highly complex and dynamic coordination of cytoskeletal remodeling in response to RTK stimulation [10,94]. A wide range of approaches in CSF-1-dependent macrophage cell lines, from microinjection of Rho family GTPases to fluorescence resonance energy transfer (FRET), have been used to determine the specific roles played by individual Rho family GTPases in macrophage motility. Cdc42 is important for sensing of CSF-1 gradients, membrane protrusions, especially filopodia and focal complex formation [85,95,96]. CSF-1-induced activation of Rac stimulates ruffling and lamellipodial spreading plus adhesion while Rho triggers retraction of the trailing edge via actomyosin contractility in bone marrow-derived macrophages (BMM) [83,84,97,98]. While the complexities and redundancies of Rho family GTPase signaling make it difficult to further tease apart their specific roles in CSF-1-induced motility or determine their upstream activators, it is apparent that PI3K activation is central to their mediation of CSF-1-induced macrophage motility. Hence, macrophage motility is more likely to be successfully targeted by inhibiting PI3K-induced PIP3 production at the top of the radiating motility signaling network in macrophages. Furthermore, p110δ is the specific PI3K isoform producing PIP3 in response to CSF-1 stimulation in macrophages and a selective p110δ inhibitor, idelalisib, is already used in the clinic to treat patients with neoplastic B cell disorders such as chronic lymphocytic leukemia [99]. Widening the clinical applications of idelalisib to inhibition of solid tumor invasion by preventing migration and invasion of TAMs in these tumors, may prove clinically useful. 

## 6. CSF-1R pY974, SFKs and Macrophage Motility

The SFKs have many roles in many different cell types. However, a key function of this family of non-receptor tyrosine kinases is the regulation of cell adhesion and motility [100,101]. Macrophages express six of the eight mammalian SFKs at some stage in their maturation process, reflecting the complex and plastic proteome of these cells. Three of the six, Src, Fyn and Yes, are broadly expressed in most cell types, but the remaining three, Hck, Fgr and Lyn, are primarily myeloid SFKs [102]. In keeping with the role of SFKs in adhesion and motility, CSF-1-induced maturation of macrophages from non-adherent precursor cells into adherent, migratory macrophages, produces marked changes in the expression of Src, Fyn and Fgr [14]. While there are specific biological effects for each of the SFKs expressed in macrophages, overlapping effects are common [103]. This was made abundantly clear when the sole phenotype of the Src-deficient mouse was osteopetrosis, due to loss of osteoclast resorptive capacity, and deficiency of both Yes and Fyn, as well as Src, were needed to produce embryonic lethality [104,105]. Indeed, the expression levels of individual SFKs may be coupled with those of other SFKs. For example, a striking reduction in Src levels is associated with increased Hck and Fgr expression in macrophages expressing a CSF-1R with a mutation in the C-terminal tyrosine (Y974F) and these macrophages have reduced motility (Figure 3) [14]. Accordingly, pan-SFK targeting inhibits CSF-1-stimulated motility and invasion in macrophages [14]. Moreover, SFK inhibition completely blocks co-migration and invasion of co-cultured macrophages and tumor cells into Matrigel in an in vitro invasion model [106]. To specifically target macrophage motility in this co-invasive behavior, it is important to identify which macrophage SFKs mediate the CSF-1 signal to motility and invasion. Similar to the ubiquitous SFKs, mice lacking myeloid SFKs, either individual or in dual combinations, did not show any defects in macrophage motility in a thioglycollate-induced peritonitis model and loss of all three myeloid SFKs was needed to uncover a motility phenotype in vivo [103,107]. In vitro studies of motility in BMM extracted from the various myeloid SFK mutant mice examined G-protein coupled receptor-mediated chemokine responses and integrin-based signaling but not CSF-1-induced motility [103]. Nevertheless, integrin-based studies revealed that both Hck and Fgr mediate macrophage adhesion signaling through α5β1 integrin during spreading on fibronectin and macrophages lacking both SFKs have reduced motility [108]. The two SFKs are redundant as the single mutant BMM counterparts do not display apparent adhesion and motility deficiencies [108]. Unsurprisingly, spreading and motility defects are more profound when all three myeloid SFKs are lost [107]. However, compensatory changes in expression of the remaining ubiquitous and myeloid SFKs in macrophages following knockout of one or more SFKs will likely confound these results and approaches to either acutely reduce the expression of individual SFKs or, alternatively, to activate specific SFKs through mutation of the negative regulatory C-terminal tyrosine or use of inducible, activatable constructs will help tease apart specific functions for individual SFKs [109]. Our understanding of which SFKs mediate CSF-1R signaling to motility in macrophages is still somewhat rudimentary but, as a group, they are key transducers of CSF-1R signaling through augmentation of phosphotyrosine-based signaling [18,46]. Both Hck and Lyn associate with the CSF-1R, Hck in a CSF-1 dependent manner and Lyn constitutively, with a notable CSF-1-induced increase in Hck tyrosine phosphorylation [14]. Consistent with this, we have examined BMM extracted from mice with a constitutive activation mutation of the Hck gene [110], and found that they move more rapidly than wild type BMM in response to CSF-1 and degrade and invade matrix significantly more efficiently than controls [111]. Considering expression levels of the other macrophage SFKs are unaltered in these Hck mutant BMM, it would appear that Hck is a major, if not the primary, SFK transducing the CSF-1R motility signal in macrophages. Importantly, a Hck selective inhibitor, RK-20449, has recently been developed and has been shown to promote tumor regression in mouse xenograft models [112,113]. Hence, inhibition of macrophage motility by targeting Hck with RK-20449 may be a viable therapeutic approach. 

## 7. Inhibiting CSF-1-induced Macrophage Motility to Prevent Tumor Invasion

TAMs contribute to the development and progression of cancer at almost every step in the complex process [114]. Moreover, chemotherapy and radiotherapy appear to further encourage TAM recruitment with negative consequences for treatment response [115]. In particular, TAMs play a critical role in encouraging tumor invasion through induction of a paracrine dialog between tumor cells and closely associated TAMs, which leads to their co-migration and invasion away from the primary tumor [56,58,59]. Consistent with this, 3D studies of mammary tumor spheroid invasion demonstrate that tumor cells are incapable of invasion into the surrounding matrix unless the spheroids are infiltrated with macrophages, indicating that macrophages trigger and support tumor cell invasiveness [106,116]. Live imaging of the tumor cells and macrophages highlights the streams of co-migrating macrophages and tumor cells as they invade the Matrigel and macrophages can be seen encouraging tumor cell migration as they follow macrophages through tunnels previously dug by those macrophages [106]. Consistent with the notion that macrophage motility regulates tumor invasion rates, hyper-motile macrophages provoke greater tumor invasion [111], while selective inhibition of macrophage motility with idelalisib or RK-20449 completely blocks tumor invasion in this co-culture spheroid model [106]. With the identification of two core CSF-1R-triggered motility pathways involving PI3K p110δ and Hck that are selectively expressed in hematopoietic cells and abundantly expressed in macrophages, we can now specifically target migratory macrophages to prevent their promotion of tumor invasion without depleting tumors of more stationary TAM populations that may be providing anti-tumoral functions.

## 8. Concluding Remarks

Macrophages and other mononuclear phagocyte-derived cells, such as osteoclasts and microglia, integrate with parenchymal cells in every tissue and organ system during early embryogenesis. As they move through tissues, they monitor their local environment and respond to perturbations in the steady state. Tumors are aberrant tissues populated with their own population of tissue-adapted macrophages, TAMs. As CSF-1 is the primary regulator of most tissue macrophage populations, including TAMs, and is also a potent macrophage chemokine, CSF-1 or CSF-1R inhibitors have been tested clinically to determine whether they could successfully block TAM recruitment. However, full CSF-1R blockade may have deleterious effects by also inhibiting and depleting anti-tumoral TAM populations. Hence, selective targeting of specific functions of tumor-promoting macrophages, such as their promotion of tumor cell invasion through co-migration of TAMs and tumor cells, might prove to be more therapeutically useful. Using a series of CSF-1-dependent, adherent macrophage cell lines expressing CSF-1R molecules with mutations in individual tyrosine residues, the primary mediators of CSF-1-induced signals regulating macrophage motility and invasive capacity were demonstrated to be a Class 1A PI3K, PI3K p110δ, and a myeloid SFK, Hck. Isoform selective PI3K p110δ and Hck inhibitors are available and may be deployed to prevent the activation of tumor invasion by co-migrating TAMs, which not only provide EGF to stimulate tumor cell motility, but also dig tunnels into the surrounding matrix through which tumor cells can escape.
